# Androgen/androgen receptor axis maintains and promotes cancer cell stemness through direct activation of Nanog transcription in hepatocellular carcinoma

**DOI:** 10.18632/oncotarget.9192

**Published:** 2016-05-05

**Authors:** Lupin Jiang, Juanjuan Shan, Junjie Shen, Yanzhou Wang, Ping Yan, Limei Liu, Wenxu Zhao, Yanmin Xu, Wei Zhu, Li Su, Jun Chen, Feifei Cheng, Hong Yao, Huicheng Xu, Cheng Qian, Zhiqing Liang

**Affiliations:** ^1^ Department of Obstetrics & Gynecology, Southwest Hospital, Third Military Medical University, Chongqing, China; ^2^ Institute of Pathology and Southwest Cancer Center, Southwest Hospital, Third Military Medical University, Chongqing, China; ^3^ Key Laboratory of Biorheological Science and Technology, Ministry of Education, Bioengineering College, Chongqing University, Chongqing, China

**Keywords:** hepatocellular carcinoma, androgen/androgen receptor axis, Nanog, cancer stem cells, CRISPR/Cas9

## Abstract

Hepatocellular carcinoma (HCC) is one of the most common and malignant cancers. The HCC incidence gets a strong sexual dimorphism as men are the major sufferers in this disaster. Although several studies have uncovered the presentative correlation between the axis of androgen/androgen receptor (AR) and HCC incidence, the mechanism is still largely unknown. Cancer stem cells (CSCs) are a small subgroup of cancer cells contributing to multiple tumors malignant behaviors, which play an important role in oncogenesis of various cancers including HCC. However, whether androgen/AR axis involves in regulation of HCC cells stemness remains unclear. Our previous study had identified that the pluripotency factor Nanog is not only a stemness biomarker, but also a potent regulator of CSCs in HCC. In this study, we revealed androgen/AR axis can promote HCC cells stemness by transcriptional activation of Nanog expression through directly binding to its promoter. In HCC tissues, we found that AR expression was abnormal high and got correlation with Nanog. Then, by labeling cellular endogenous *Nanog* with green fluorescent protein (GFP) through CRISPR/Cas9 system, it verified the co-localization of AR and Nanog in HCC cells. With *in vitro* experiments, we demonstrated the axis can promote HCC cells stemness, which effect is in a Nanog-dependent manner and through activating its transcription. And the xenografted tumor experiments confirmed the axis effect on tumorigenesis facilitation *in vivo*. Above all, we revealed a new sight of androgen/AR axis roles in HCC and provided a potential way for suppressing the axis in HCC therapy.

## INTRODUCTION

Hepatocellular carcinoma (HCC) is one of the most common cancers with a high rate of mortality. Indeed, sound scientific evidences showed that the incidence of HCC has a prominent gender prone to the male, and the ratio of male to female ranges from 2.5 to 11:1. Several studies supported the androgen/androgen receptor axis as a pivotal factor in this bias [1, 2]. Androgen receptor (AR) is a 110 kDa transcription factor, which plays a role in regulating the expression of target genes after being activated by androgen [3]. The axis had been demonstrated to be involved in many kinds of cancer-related processes, like facilitating cancer cell growth and modulating cell cycle through TGFβ1 or β-catenin pathways, respectively [4–7], and participating in cellular growth and proliferation associated with FOXA1/2 [8–10]. However, the underneath mechanisms of the axis in the hepatocarcinogenesis gender disparity are still largely unknown.

Cancer stem cells (CSCs) are a subgroup cancer cells with high self-renewal, extensive proliferation, and strong tumorigenesis capacity, which had been considered as the initial cells in cancer development [11]. Recently studies demonstrated that androgen/AR axis participated in CSCs regulation of prostate cancer. However, these effects were pleiotropic, as well the complicated mechanisms remains poorly understood and need further investigated [12–14].

In our previous work, we had demonstrated the pluripotency factor Nanog can be a reliable CSCs marker in HCC. It also participated in maintaining stemness of CSCs [15]. Although the downstream regulation networks of Nanog have been well studied, the knowledge about regulating Nanog in HCC remains limited [16–18]. Bioinformatics analysis showed there are putative AR binding sites in *Nanog* promoter. Therefore, we wondered that if the androgen/AR axis had effect on stemness maintenance of HCC cells through the Nanog related pathway.

In this study, we investigated the effect of androgen/AR axis on HCC cells stemness and then to elucidate the mechanism behind it. For this purpose, firstly, we demonstrated the AR was highly expressed in hepatocarcinoma than the peritumorial tissues, and androgen can promote stemness of HCC cells. We also found the Nanog expression was coincidence with AR in hepatocarcinoma tissues. Then, by labeling endogenous *Nanog* with GFP via CRISPR/Cas9-induced homology-directed repair way in HCC cells, it confirmed the AR and Nanog are exactly co-localization in these cells. Further data revealed that androgen/AR axis can increase *Nanog* expression by directly binding to its promoter, and promote HCC cells stemness and tumorigenesis. This effect can be abrogated by AR degradation enhancer or androgen deprivation. Thus, our findings revealed a new sight of androgen/AR role in hepatocarcinogenesis through affecting cancer cells stemness and provided evidence for this axis suppression in HCC therapy.

## RESULTS

### AR is highly expression in HCC and co-localization with Nanog in HCC tissues

**Figure 1:**
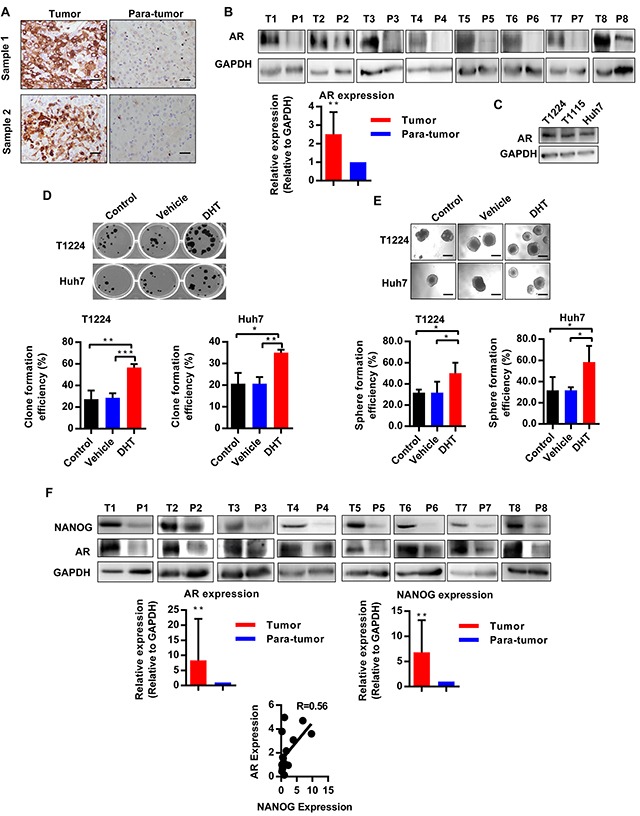
AR is highly expressed in hepatocarcinoma and is associated with expression of Nanog **A.** Immunohistochemical staining of AR expression in hepatocarcinoma and peritumoral tissues (para-tumor). Scale bars, 100 μm. **B.** Western blotting analysis the expression of AR in hepatocarcinoma (T) and corresponding peritumoral (P) tissues, GAPDH as the internal reference, n=8. **C.** AR expression in HCC cells T1224, T1115 and Huh7. **D-E**. Clone and sphere formation efficiency of HCC cells after treatment with DHT, or DMSO as vehicle. Data was presented as means ± SD of three independent experiments. **F.** Nanog and AR expression in hepatocarcinoma (T) and corresponding peritumoral (P) tissues. The data showed 8 pair out of 16 pairs. R value is correlation of AR and Nanog expression under correlation analysis by gray value normalized to GAPDH, n=16. p<0.05(*), p<0.01(**) and p<0.001(***).

To investigate the role of androgen/AR axis in HCC, we firstly detected AR expressions in 8 pairs of HCC and corresponding peritumoral tissues. Immunohistochemistry and Western blot assays showed that the AR did exist in hepatocarcinoma tissues, and its expression is significantly higher than the corresponding peritumoral counterparts (Figure 1A and 1B). Furtherly, we also found that AR was generally expressed in primary HCC cells T1115, T1224 and the HCC cell line Huh7 (Figure 1C).

Previous studies demonstrated that CSCs played a vital role in tumorigenesis. To identify there is a relationship between androgen/AR axis and CSCs in HCC, we utilized Dihydrotestosterone (DHT), a physiologic agonist of AR, to treat the primary T1224 and Huh7 cells. Results showed the treatment of HCC cells with DHT could increase clone and sphere formation efficiencies (Figure 1D and 1E), suggesting that the androgen/AR axis may plays a role in promoting stemness of HCC cells.

Our previous study had identified that credible stem cell marker Nanog took the core position in CSCs stemness of HCC. And it has been reported that androgen could increase Nanog expression in prostate cancer [19]. These data inspired us to wonder whether the effect of androgen/AR axis on stemness of HCC cells was Nanog depended. To verify it, firstly, we detected the AR and Nanog expression in 16 HCC samples. As results, both AR and Nanog were highly expressed in HCC, as compared to the corresponding peritumoral tissues, and their expression got exactly consistent (Figure 1F, Supplementary Figure S1), which connected the androgen/AR axis with Nanog in the HCC tissues.

Above all, the results demonstrated that AR was abnormal highly expressed and co-localization with Nanog in HCC tissues, which was associated with stemness of HCC cells.

### AR expression gets consistent with endogenous Nanog labeled by CRISPR/Cas9 system in HCC cells

**Figure 2:**
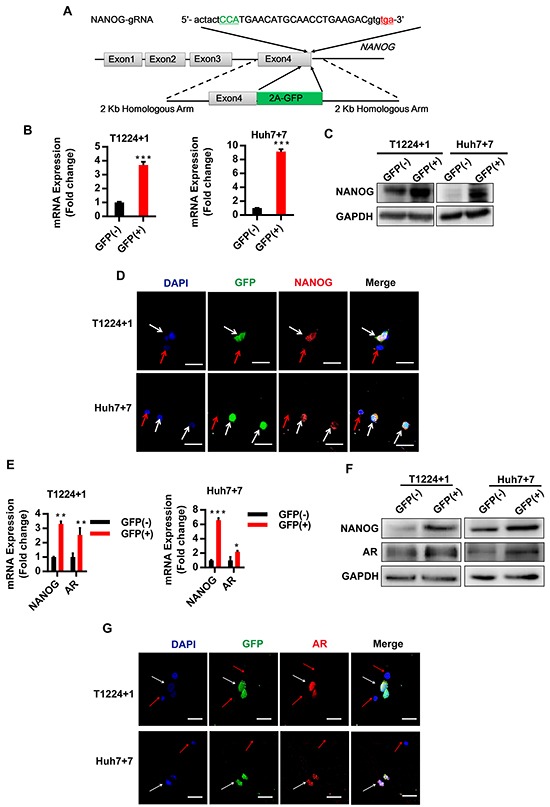
AR is co-localization with Nanog in HCC cells based on GFP labeled Nanog cells by CRISPR/Cas9 system **A.** The schematic of the Nanog labeled CRISPR/Cas9 system: the green capital refers to Cas9 recognize protospacer adjacent motifs (PAM) sequence, and the red lowercase refers to *Nanog* termination codon. *Nanog* exon4 without termination code jointed with 2A-GFP sequence, and each length of homologous arm is 2Kb. **B-C.** RT-qPCR and Western blot were performed to measure the mRNA of Nanog in GFP (+)/(−) cells of two labeled clone cells, qPCR values are normalized to GAPDH and represent the mean ± SD of triplicate samples. **D.** The confocal image of Nanog and GFP co-localization in T1224+1 and Huh7+7 cells, blue: DAPI, green: GFP, red: NANOG, white arrow: co-localization positive, red arrow: co-localization negative. **E-F.** RT-qPCR and Western blot detected the expression of Nanog and AR in two clone GFP (+)/(−)cells. **G.** The confocal images of T1224+1 and Huh7+7, blue: DAPI, green: GFP, red: AR, white arrow: co-localization positive, red arrow: co-localization negative. Scale bars, 100μm, p<0.05(*), p<0.01(**) and p<0.001(***).

Then, to study the endogenous *Nanog* expression and changes in different conditions, we used CRISPR/Cas9 system to label *Nanog* with green fluorescent protein (GFP), expecting that the fluorescent of GFP can represent the *Nanog* expression directly and accurately (Figure 2A). PX330, a plasmid for expression of Cas9, combined with a chimeric guide RNA (gRNA) coding sequence in one vector backbone were used in this study [20]. And the Nanog gRNA was inserted in it to generate *Nanog* gene target CRISPR/Cas9 vector PX330-Nanog-gNRA. After checking our CRISPR/Cas9 system accuracy and effectiveness in HEK293FT cells (Supplementary Figure S2), we transfected PX330-Nanog-gRNA vector and the donor plasmid into T1224 or Huh7 cell lines simultaneously to generate *Nanog* labeled HCC cells. Verified by PCR, restriction enzyme digestion and Sanger sequencing, we got some correct single clones (Supplementary Figure S3), and we randomly chose two independent clones, as T1224 clone 1 (hereafter T1224+1) and Huh7 clone 7 (hereafter Huh7+7), for followed experiments.

Next, we identified Nanog expressions between GFP (+) and (−) cells of the two clones. As predicted, the expressions of Nanog in GFP (+) cells were significantly higher than the GFP (−) cells both at mRNA and protein level (Figure 2B and 2C). Also, the confocal images confirmed that the GFP and Nanog were co-localization, as GFP (+) cells were Nanog positive and GFP (−) cells were Nanog negative (Figure 2D). Then, we verified the proliferation, self-renewal and tumorigenesis abilities to confirm different stemness between the GFP (+) and (−) cells *in vitro* and *in vivo*. From the clone and sphere formation assays, it showed the GFP (+) cells generated obviously more clones and spheres than the GFP (−) cells (Supplementary Figure S3F and S3G). Mouse subcutaneously transplanted tumor model showed that the tumor formation rate were 4/6 vs 2/6 between GFP (+) and (−) cells from T1224+1, and 4/6 vs 1/6 from Huh7+7 (Supplementary Figure S3H). These data indicated that GFP fluorescence can exactly represent Nanog expression in the labeled cells.

With these Nanog labeled single clone cells, we went on to clarify the relationship between AR and Nanog. As the results, there were high expression of Nanog and AR in GFP (+) cells, compared to the GFP (−) cells at mRNA or protein levels. Also, their expression tendency got consistency (Figure 2E and 2F). Then, immunofluorescence assay confirmed the co-localization of AR and Nanog, as AR positive signals were detected in GFP (+) cells, but not in GFP (−) cells (Figure 2G).

Above results demonstrated that we obtained the correct endogenous *Nanog* labeled HCC cells by using CRISPR/Cas9 gene target system, and Nanog expression was indeed consistent with AR in HCC cells.

### Androgen/AR axis stimulates Nanog expression and promotes HCC cell stemness

Since our data showed Nanog was co-localization with AR and their expression got correlation both in HCC tissues and cells, we wondered if there was inner connection between these two factors. And, because AR is a transcription factor and the androgen/AR axis had been reported to regulate many gene expressions, we went to identifying if the axis also had effect on Nanog expression in HCC cells.

**Figure 3:**
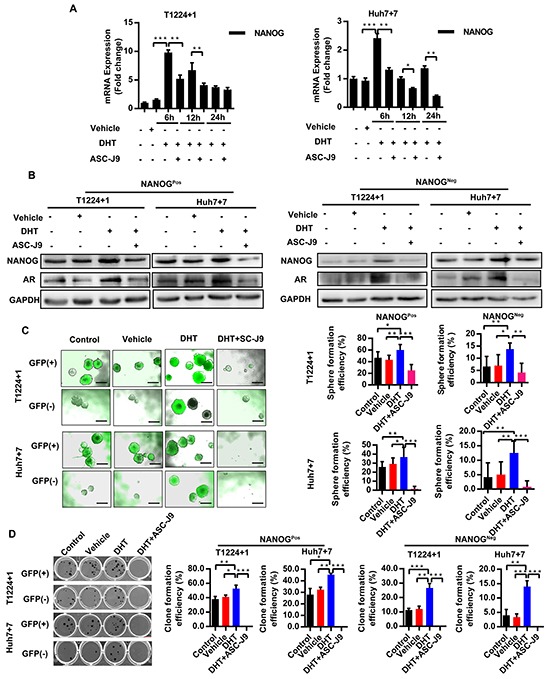
Androgen/AR axis stimulates Nanog expression and promotes HCC cells stemness **A.** Level of Nanog mRNA was detected at the different time points after treatment with DHT together with or without ASC-J9 in T1224+1 and Huh7+7 cells. Values are normalized to GAPDH and represented the mean ± SD of triplicate samples. **B.** Protein levels of AR and NANOG expression was measured in Nanog^Pos^ and Nanog^Neg^ cells from T1224+1 and Huh7+7after treatment with DHT and with or without ASC-J9 for 24h. **C-D.** Clone and Sphere formation efficiencies of Nanog^Pos^ and Nanog^Neg^ cells from T1224+1 and Huh7+7 after treated with DHT, or with or without ASC-J9. Data was presented as means ± SD of three independent experiments. The clone and sphere formation efficiency were calculated as the percentage of cell clones or spheres number divided the total cells number that seed in each well. Scale bars, 200μm, p<0.05(*), p<0.01(**) and p<0.001(***).

T1224+1 and Huh7+7cells were treated by the DHT, an AR physiological activator, with or without ASC-J9, an AR degradation enhancer that specially accelerates it degeneration, which has little side-effect on other pathways compare to traditional AR antagonist like Bicalutamide [21–23], to activate or inhibit the axis. After testing different concentrations of DHT and ASC-J9 on the cells viability by MTS assay at different time points (Supplementary Figure S4A), we chose the appropriate concentration of each drug as 10nM for DHT (normal human serum androgen concentration) and 5μM for ASC-J9 for further experiments. Our results showed that mRNA levels of both AR and Nanog increased after treatment with DHT, whereas this stimulatory effect was counteracted by ASC-J9 (Figure 3A and Supplementary Figure S4B). In addition, we noticed that AR mRNA did not decreased as remarkably as Nanog in the ASC-J9 treated group, which attributed to ASC-J9 promotes AR protein degeneration but scarcely affects its transcription. This indicated that the androgen/AR axis can stimulate Nanog expression. Similar results were also confirmed at protein level. To check if the axis triggers the same effect on both GFP (+) (hereafter Nanog^pos^) and GFP (−) (hereafter Nanog^neg^) cells, we sorted and treated them separately. As expected, in DHT treated groups, the Nanog expression increased in both Nanog^pos^ and Nanog^neg^ cells. The addition of ASC-J9 attenuated the DHT effect and reduced the expression of AR and Nanog in both groups (Figure 3B).

To verify the biological effect of androgen/AR axis on Nanog can indeed promote cells stemness, the clone and sphere formation assays were then taken in T1224+1 and Huh7+7cells. Our results showed that treatment with DHT generated more clones and spheres, and this effect was attenuated in by ASC-J9 (Figure 3C and 3D). Drug resistance experiment also demonstrated DHT can increase HCC cells resistance to the Cisplatin (Supplementary Figure S4C). In addition, we found that the androgen/AR axis made a similar impact on both Nanog^pos^ and Nanog^neg^ cells. Interestingly, we found there were some spheres turned to Nanog^Pos^ in the Nanog^Neg^ groups. This phenomenon indicated the axis could not only maintain the stemness of Nanog^Pos^ cells, but also had effect on HCC cells dedifferentiation by turning Nanog^Neg^ cells from non-cancer stem cells to stem-like cells. To further confirm this conclusion, we examined expression of stemness markers as Oct4 and Sox2 in the Nanog^neg^ cells under the different treatments. The results showed that both Oct4 and Sox2 were increased as well as AR was up-regulated in the DHT treated groups (Supplementary Figure S4D). However, since the dedifferentiation processes are comprehensive, mechanism and network behind it needed further studies. All together, we demonstrated that androgen/AR axis could stimulate Nanog expression in both Nanog^Pos^ and Nanog^Neg^ cells, and maintains stemness in Nanog positive cells and promotes Nanog negative cells to get stem cell like characters.

### AR increases Nanog expression through directly binding to its promoter and inducing the promoter activity

Next, we investigated the mechanism of androgen/AR axis on regulation of Nanog expression. Since AR is a nuclear transcription factor and Nanog was up-regulated by DHT at both mRNA and protein levels, we wondered if AR could regulate Nanog expression by directly binding to and activating its promoter in HCC cells.

**Figure 4:**
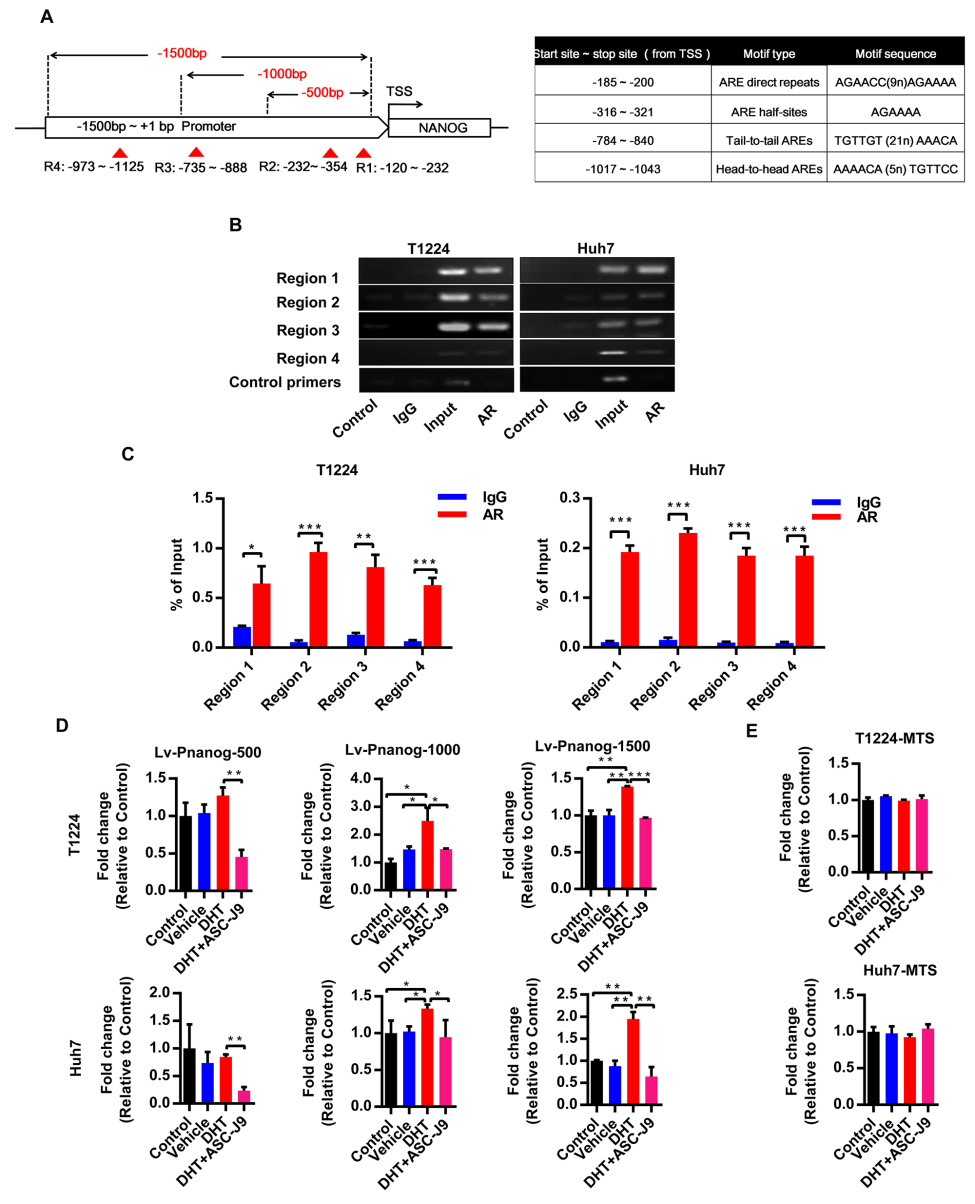
Androgen/AR axis stimulates Nanog transcription activity by binding to its promoter **A.** The schematic of Nanog promoter regions from transcriptional start site (TSS) to −1500bp and the exactly motif sequence. Dotted line: different length of *Nanog* promoter luciferase report fragment to the TSS, red triangle and region 1-4 (R1-4): the predict AR binding motif sites in the Nanog 1500bp promoter. ARE: androgen responsive element. **B-C.** ChIP assay for AR binding to Nanog promoter in T1224 and Huh7 cells. Control and IgG were used as sample negative control, Input as sample positive control. Data normalized to Input and represented the mean ± SD of triplicate samples **D.** Different lengths of Nanog promoter luciferase activity after treatment of cell with 10nM DHT with or without 5uM ASC-J9. **E.** Cell viability after treatment of cells with 10 nM DHT with or without 5uM ASC-J9 by MTS assay. Data was presented the mean ± SD of triplicate samples. p<0.05(*), p<0.01(**) and p<0.001(***).

Previous studies have identified four canonical types of AR binding motif [24, 25]. Moreover, more motifs derived from the classical types with high affinity to AR also have been found recently [26–28]. Based on this information, we examined canonical and non-canonical AR binding motifs in the *Nanog* promoter by bioinformatics analysis. We found that there are some AR special and high affinity binding elements derived from the classical ones in the *Nanog* promoter region from transcription started site (TSS)+1 to −1500bp (Figure 4A). Then we verified the bindings by Chromatin Immuoprecipitation (ChIP). The results showed that AR could directly bind to the indicated *Nanog* promoter regions both in T1224 and Huh7 cells (Figure 4B and 4C).

Next, to confirm if this binding could increase the promoter activity, we chose different lengths of *Nanog* promoter from TSS to −500/−1000/−1500 bp upstream to constructed luciferase report lentivirus (named Lv-PNanog-500/1000/1500) respectively (Figure 4A). Our results showed that DHT could increase *Nanog* promoter activities, especially in −500~-1500 bp regions in both T1224 and Huh7 cells, and degeneration of AR by ASC-J9 could reduce the activities in all different regions (Figure 4D). We also found that the effect of DHT on activation was not significant in the first 500bp region, but ASC-J9 could reduce the activity. This result suggested there were some background bindings between them, and effects of the axis on Nanog promoter are cumulative. Additionally, we found that all these treatments had no obvious influence on cell viability by MTS assay (Figure 4E). Taken together, we revealed that androgen/AR promotes Nanog expression through binding to its promoter directly.

### Androgen/AR axis takes effect on HCC cells stemness via Nanog

**Figure 5:**
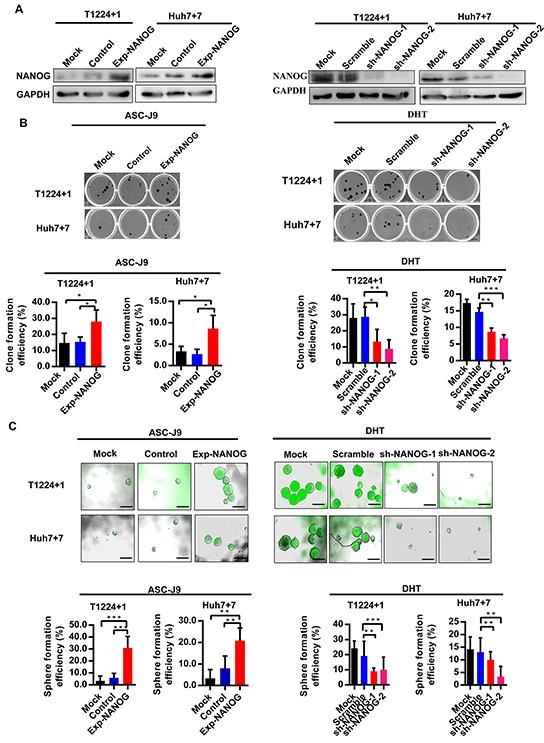
Effect of Androgen/AR axis on stemness of HCC cells is depended on Nanog **A.** Western blot analysis of the Nanog expression in T1224+1 and Huh7+7 Nanog over-expression or knock-down cells. Control: Nanog over-expression control lentivirus; Exp-NANOG: Nanog over-expression lentivirus; Scramble: sh-NANOG control lentivirus; sh-NANOG-1 and sh-NANOG-2: sh-NANOG lentivirus. **B-C.** Clone and sphere formation efficiencies in T1224+1 and Huh7+7 cells with overexpression of Nanog after treatment with ASC-J9 and in T1224+1 and Huh7+7 cells with knockdown of Nanog expression after treatment with DHT. Data was presented the mean ± SD of triplicate samples. Scale bars, 200μm, p<0.05(*), p<0.01(**) and p<0.001(***).

Further, to identify whether the androgen/AR axis promotes HCC cells stemness indeed through up-regulating Nanog, we next overexpressed or knocked down *Nanog* in T1224+1 and Huh7+7 cells by lentivirus, respectively (Figure 5A). Then the clone and sphere formation assays were taken again to verify whether the DHT or ASC-J9 could still promote or inhibit the stemness in these *Nanog* disturbed cells. As the results, in the *Nanog* knock-down cells, DHT could no longer stimulate the clone or sphere formation, and the *Nanog* over-expression cells counteracted the stemness inhibit effect of ASC-J9 (Figure 5B and 5C). These results indicated the androgen/AR axis promoted HCC cells stemness through regulating Nanog.

### Androgen/AR axis stimulates tumorigenesis *in vivo*

**Figure 6:**
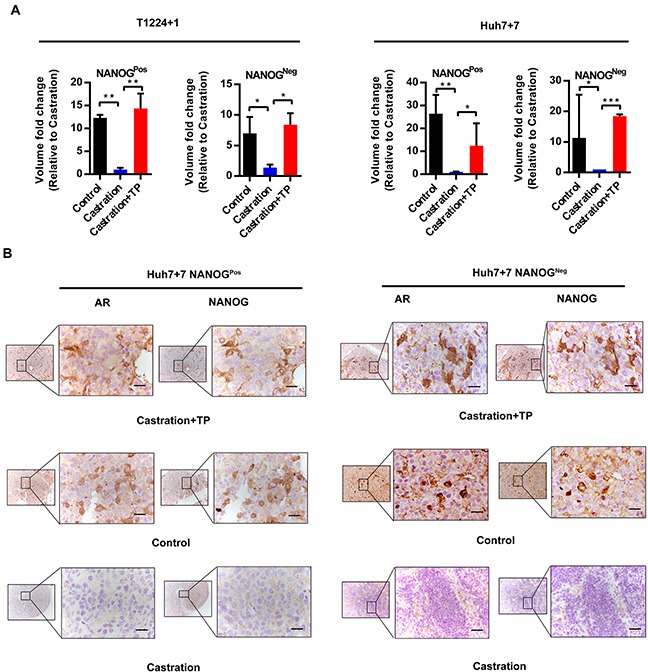
Androgen/AR axis promotes oncogenesis of HCC cells in vivo **A.** Nanog^Pos^ and Nanog^Neg^ cells from T1224+1 and Huh7+7 were subcutaneously implanted in male nude mice. Animals were derived as groups of control (no treated), castrated or castrated supplemented with testosterone propionate (TP). The tumor volume was measured and presented as mean ± SD of 6 mice per group. P<0.05(*), p<0.01(**) and p<0.001(***). **B.** Immunohistochemistry staining of AR and Nanog in continuous sections from subcutaneous transplantation tumor derived from Huh7+7 cells. Scale bars, 100μm.

Then, we took subcutaneous xenografts model in nude mice to confirm the effect of androgen/AR axis on tumorigenesis. Before tumor cells injection, male nude mice were castrated or not, then the castrated ones were supplemented with or without testosterone propionate (TP) during tumor growing. As the result of T1224+1 and Huh7+7 cells tests, tumor volumes of both Nanog^Pos^ and Nanog^Neg^ cells were obviously greater in control or the castrated supplemented with TP groups than the only castratedones (Figure 6A). This confirmed that androgen/AR axis could maintain or promote stemness of Nanog^Pos^ and Nanog^Neg^ cells respectively. Additionally, above result was verified by immumohistochemical staining of the xenograft tumor serial sections, in which the AR and Nanog expression in control and castration supplemented TP groups were obviously higher than the castration ones, and the expression of AR and Nanog reduced significantly in the castration groups. Also the expression of Nanog in accord with AR in all groups (Figure 6B and Supplementary Figure S5). These *in vivo* experiments further confirmed that the androgen/AR axis could promote HCC tumorigenesis.

## DISCUSSION

The gender dimorphism of HCC morbidity between men and women has been observed and confirmed for a long time, and sex hormone pathways are considered as important accomplices. Previous studies had revealed the estrogen as a protect factor in this accident [29], but the effect of androgen/AR axis seems pleiotropic [30]. In recent years, increasing evidences have considered androgen/AR axis as a motivator for tumorigenesis in prostate, liver and some other organs [31, 32], which enriched our understanding of HCC gender disparity phenomenon, but the exact mechanisms are still largely unrevealed.

With the hypothesis that androgen/AR axis was involved in hepatocarcinogenesis, we firstly confirmed AR was highly expressed in HCC tissues. Furthermore, with the clue that recent studies reported androgen/AR axis increased Nanog expression in prostate cancer cells, and our previous study has identified Nanog as an important stemness regulator in HCC cells, we wondered whether the axis could impact on HCC cells stemness.

Then, by labeled *Nanog* with GFP through CRISPR/Cas9 based knock-in method, we obtained the Nanog labeled HCC single clone cells, and it clearly showed that AR was co-localization with Nanog, as well as their expression got coincidence in HCC cells.

Subsequently experiments demonstrated that AR could directly bind to the *Nanog* promoter to stimulate its expression, promote the stemness of HCC cells and trigger oncogenesis. In addition, we found the AR binding motifs sequence in HCC cells were a bit different from the canonical types, and it could be attributed to the binding motif variants or tissue specificity [33, 34], which need further study. Moreover, we noticed that under the androgen stimulating, the Nanog^Neg^ cells could turn to be Nanog^Pos^. Since others and our previous studies had demonstrated Nanog gene is a key pleuripotent regulate factor in HCC CSCs stemness maintenance and played a role in non-CSCs dedifferentiation, as the Nanog^neg^ cells turned to Nanog^pos^, it implied a dedifferentiation phenomenon in these cells [15, 35]. However, because of the dedifferentiation processes of cancer cells are complicated and include cooperative networks, our finding may provide a promising phenomenon for following investigation.

In this study, we revealed the relationship between androgen/AR axis and HCC cells stemness regulation. Moreover, judging from the current situation of liver cancer, a certain number of patient origins from the HBV infection, especially in developing countries like China, and the biological properties of HBV have already been reported to associate with AR effect in hepatocarcinogenesis recently [36, 37], it is significant to recover the relationship among the androgen/AR axis, HCC stem cell, and HBV.

Considering that androgen/AR axis plays an important role in hepatocarcinogenesis, combination of traditional therapies with the androgen deprivation or antagonist to AR might be promising therapeutic strategies in HCC treatment. In the last decades, some groups indeed tried anti-androgen therapy to prevent HCC in clinical trials, but got limited curative effect [38, 39]. The reason may be in keeping with the relapse of prostate cancer after androgen deprivation therapy (ADT). On the one hand, the androgen can be complemented by other organ (eg. adrenal) or tissues [40–43]. And on another hand, the androgen/AR pathway is widely regulated, and can be activated vicariously [44, 45]. Considering the variable hormone conditions and AR regulations in particular tissues, the strategy to decrease the androgen in tumor microenvironment or to inhibit its binding to AR may be promising ways in treatment of such male bias cancers.

## MATERIALS AND METHODS

### Tissue samples and cell culture

Fresh tumor specimens were obtained from patients underwent surgical resection of primary HCC at the Institute of Hepatobiliary Surgery, Southwest Hospital, Third Military Medical University. All patients with informed consent from according to protocols approved by the Institutional Review Board of the Southwest Hospital, Third Military Medical University.

Human primary hepatic carcinoma cells T1115 and T1224 were obtained from the HCC patients to establishing lines as described previously [15], while HEK293FT from our lab keeping and Huh7 were purchased from the Shanghai Cell Collection (Shanghai, China). Cells were cultured in Dulbecco's modified Eagle's medium (DMEM) supplemented with 10% fetal bovine serum (GIBCO-BRL), 100 U/mL of penicillin sodium and 100 mg/mL of streptomycin sulfate (Invitrogen Life Technologies), at 37°C in a 5% CO2 atmosphere.

### Endogenous *Nanog* labeled HCC cells by CRISPR/Cas9 system

Constructing Nanog labeled HCC cells by CRISPR/Cas9 system performed as described in supplementary materials and methods. The gRNA and primers sequence were listed Supplementary Table S1 and S2.

### Cell transfection and infection

When cell confluence reached 70%-80%, 0.25ug PX330-NANOG-gRNA plasmid combined with or without 0.15ug NANOG-2A-GFP homogeneous arm vector were transfected into cells by Effectene Transfection Reagent (Qiagen), respectively. GFP fluorescent were examined by fluorescence microscope at 72h after transfected.

For Nanog over-expression or knock-down experiments, 10 MOI of Control/ Exp-NANOG/ Scramble/shNANOG-1/shNANOG-2 lentivirus were transfected to each cell, respectively.

### Endonuclease enzyme digested and T7E1 assay

Endonuclease enzyme digested and T7E1 assay performed as described in supplementary materials and methods.

### Fluorescence activated cell sorting

GFP (+) and (−) cells of all transfected cells were sorted by FACS Aria II (BD Biosciences) as threshold were 5% for each cell. Single of T1224 or Huh7 labeled GFP (+) cell was seed into each well of the 96-well plate for expand culture.

### RNA extraction and RT-qPCR analysis

1×10^6^ cells were prepare for analysis, RNA was extracted by Eastep Super RNA extract kit (Promega) and reversed by Advantage® RT-for-PCR Kit (Takara), the standard RT-qPCR performed with CFX96^TM^Real-Time system (BIO-RAD). The primers were listed in Supplementary Table S3. All experiments were performed in triplicate.

### Western blotting assay

For protein extraction, whole-cell lysates collected from 1×10^6^ cells were used per lane. Briefly, cells were washed by ice PBS and lysed with cell lysis buffer (Thermo Fisher scientific), the tissues were smashed and the proteins were extracted by tissue lysis buffer (Thermo Fisher Scientific). Western blot assay was performed as previously described. Antibodies used were: anti-Nanog (Cell Signaling Technology), anti-AR (N-20, Santa Cruz Biotechnology), anti-GAPDH (Cell Signaling Technology), anti-Oct4 (Cell Signaling Technology) and anti-Sox2 (Abcam).

### Cell treatment

For hormone treatment, the cultured cells were washed twice with PBS and changed into serum-free medium for hormone deprived 24h before treating, to avoid the factors in plasma affect the AR activity. Then 10nM DHT (Dr. Ehrenstorfer GmbH) with or without 5μM ASC-J9 (MedChem Express) dissolved in DMSO (vehicle) were add into the medium, respectively. For Western blot, cells were treated for 24h, and for RT-PCR, cells were treated as mentioned time points before harvested.

### Sphere formation assay

For GFP (+)/(−) cells identification, 10 single GFP(+) or (−) cells of T1115+2/T1224+1/Huh7+7 were sorted by FACS into 96 ultra-low attachment surface plate (Corning) within DMEM/F12 (Invitrogen) medium supplemented with B27 (Invitrogen), HGF, bFGF, and EGF (Prepro Tech). For hormone treatment, T1224+1/Huh7+7 GFP (+)/(−) or lentivirus infected cells per well in DMEM/F12 only supplemented with B27 besides the indicated drugs to excluding the cytokines interferes. Considering the hard growth condition, 20 cells were sorted into each well. All mediums were appended every third days, and cells treated total for 14 days before analysis.

### Clone formation assay

Clone formation assay were done in 24-well plates, 50 indicated cells were sorted and seeded by FACS. For GFP (+)/(−) cells verification, the 10% FBS DMEM cultured for 14 days. For hormone treatment, 24h after seeded in, the medium were changed into DMEM with low FBS (2%), and added with or without the indicate regents. Medium replaced every two days and treated for total 14 days.

### Immunohistochemistry staining

For transplantation tumors, the tumor tissues were fixed in 4% paraformaldehyde immediately after take out from the mice. Then gone through embed, section, and conventional immunohistochemical process. The pieces incubated with AR (N20, Santa Cruz) or Nanog (Cell Signaling Technology) primary antibody respectively in 4°C for 16h and second antibody (DaKo) in 37°C for 0.5h before detected. Sections were visualized under the microscope (Olympus) and images were captured by the camera linked to a computer with the corresponding magnification.

### Immunofluorescence assay

Cells were seed into the 24 well plates that preloaded with glass slides and cultured for 12h, then fixed cell slides with 4% paraformaldehyde for 30min and perforated by 0.3% Triton (Sigma Aldrich) for 10 min. After blocked with 10% BSA (Sigma Aldrich), the slides incubated with indicated first antibodies respectively for 16h in 4°C and second antibody Alexa Fluor^®^ 568 (Thermo Fisher Scientific) for 1h in room temperature, then treated with DAPI for 15min and mounted by 40% glycerinum before exam. The immunofluorescence detected by confocal microscopy (Carl Zeiss Jena).

### Chromatin immunoprecipitation (ChIP) assay

For ChIP analysis, experiment was done according to the instruction manual of EZ-CHIPTM kit (Merck Millipore). Briefly, 1×10^7^ of T1224 and Huh7 cells were treated with 10nM DHT for 24h before harvested, then the cells were crosslinked with 1% formaldehyde for 15 min at room temperature. After cell lysis, the chromatins were subjected to sonication and fragmented into 250-500bp. Then, protein–DNA complexes were immunoprecipitated by anti-AR or control anti-IgG antibodies at 4°C for overnight with rotation. After washing and reversal of crosslinks, the immunoprecipitated and input purified DNA followed by qualitative and quantitative PCR with primers list in supplementary table S4, respectively.

### Luciferase reporter assay

The luciferase backbone lentivirus was modified from our previous study [15] to be inserted with different lengths of *Nanog* promoter regions from the TSS (−500bp, −1000bp, −1500bp). Three days after transfection, the cell were seed into 96 well plate, starved in FBS free medium for 24h and treated by vehicle, DHT with or without ASC-J9 respectively for another 24h. Luciferase activities detected with Luciferase Reporter Assay System (Promega). Each experiment repeated for triplicate.

### MTS assay

Cell viability was detected by MTS Cell Proliferation Assay kit (Promega). After starved in FBS free medium for 24h, cells treated by indicated compound for another 24h or 48h as mentioned respectively. For drug resistance experiment, cells treated by 5ug/ml cisplatin (Sigma Aldrich) with or without 10nM DHT for 48h before detected. Each experiment repeated for triplicate.

### Animals studies

We performed all animal experiments in accordance with the “Guide for the Care and Use of Laboratory Animals” prepared by the National Academy of Sciences and the institutional ethical guidelines from the Animal Ethics Committee of the Third Military Medical University. All mice were maintained in pathogen-free conditions at the animal facility of Third Military Medical University.

For GFP (+)/(−) cells verified experiment, 5×10^3^ of T1224+1 or Huh7+7 cells were sorted by FASC, and mixed with Matrigel (cell suspension volume vs Matrigel as 2:1) (BD Biosciences), then injected into two flanks of the 5-week-old male NOD/SCID mice, respectively. Each group with 3 mice and 6 transplant sites, tumors grew for total two months after injected.

For hormone treated study, the male nude mice with or without castrated at 5 weeks of age, one castrated group were intramuscular injected testosterone propionate (25mg/kg, once a week), and the other group was not, each group included 6 mice. One week after the operation, 5×10^3^ GFP (+)/(−) of T1224+1 or Huh7+7 cells mixed with Matrigel (2:1) were implanted subcutaneously of mice. 15 days after tumors established, the mice were sacrificed and tumor volumes were measured with the calculated formula as volume= (length×width^2^)/2.

### Statistical analysis

Statistical tests were done by Graph Pad Prism v 6.00 (Graph Pad Software), each experiment was repeated triplicate to determine mean standard error, and one-way ANOVA or t-test performed with normalization to control analyses to obtain P-values, as P<0.05 (*), P<0.01(**) and P<0.001(***) considered significant. Relative analysis of AR and Nanog expression was calculated with Pearson correlation coefficient formula by SPSS 13.0.

## SUPPLEMENTARY MATERIALS FIGURES AND TABLES


