# Supplementary material for: Androgen/androgen receptor axis maintains and promotes cancer cell stemness through direct activation of Nanog transcription in hepatocellular carcinoma
Source: Oncotarget. 2016 May 5;7(24):36814–28. doi: 10.18632/oncotarget.9192 (PMC5095041; doi:10.18632/oncotarget.9192)
Supplement: Supplementary file 1 [file oncotarget-07-36814-s001.pdf]

SUPPLEMENTARY MATERIALS AND METHODS

Supplementary results

Constructed and verified the GFP labeled endogenous *Nanog* cells by CRISPR/Cas9 system

PX330, a plasmid with the Cas9 expression, combined with a single guide RNA (gRNA) targeted to the sequence just before *Nanog* termination codon (hereafter, called Ng, the corresponding plasmid contained Ng called PX330-Ng), to generate a double strand break (DSB) for knock-in. Then, the GFP along with a preceding 2A-peptide coding sequence, ligated with a 2A-GFP vector flanked by 2 kb homogeneous sequence around *Nanog* termination codon by PCR and Gibson clone (Figure 2A, Supplementary methods), to generate a donor plasmid for homologous recombination. While GFP fusing to *Nanog* at the nucleotides level, two separate proteins were synthesized simultaneously by way of ribosomal skipping with the help of 2A-peptide. Because there was no promoter in GFP coding sequence, it could only be transcribed with Nanog after properly integrated.

To check the correct of our CRISPR/Cas9 system, firstly, we verified the gene target site with human embryonic kidney 293FT (HEK293FT) cells. The transfected cell DNA were amplified with verify primer1 (Supplementary Figure S2A, Supplementary Tables S1) by PCR, then the products digested by T7 endogenous I (T7EI) and under Sanger sequencing, respectively. As the results, only the group transfected with PX330-Ng can be digested (Supplementary Figure S2B), because of *Nanog* sequence in it had been cut by CRISPR/Cas9 system and repaired by NHEJ (Non-Homologous End Joining) to generated "molecular scars" around the repaired alleles, which is the special substrate for T7EI [1]. Also, Sanger sequencing showed the "molecular scars" were right around the target sites (Supplementary Figure S2C.).

Then, we transfected HCC cell line Huh7, and primary HCC cells T1224 with PX330-Ng and donor plasmid simultaneously to generate stable labeled cell lines. The single GFP (+) labeled cells were sorted into the 96 well plates by fluorescence activated cell sorter (FACS). After expending culture, the cells whole genome DNA were amplified, and the products to be digested by restriction enzyme and then to be sequencing. For PCR verify, we used the verified primers2 (Supplementary Figure S3A and Supplementary Table S1), of which the up primer is binding to *Nanog* coding sequence out of the donor plasmid and the down primer is special binding to the plasmid GFP sequence, so, only the GFP labeled *Nanog* sequence can be amplified. Then the PCR products were digested by endonuclease Hind III. In the corrected targeted *Nanog* sequence, there are three particular bands that could not be found in the control plasmid (Supplementary Figure S3B). Then, the Sanger sequencing confirmed the correct of the recombination fragments (Supplementary Figure S3C).

Finally, we got some correct labeled single cell clones (data not shown), and random chose four independent clones as T1224 clone 1, clone 5 (here after T1224+1, T1224+5), Huh7 clone 7, clone 30 (here after Huh7+7, Huh7+30) for followed experiments. Firstly, we identified Nanog expression between GFP (+) and (-) cells, as predicted, the expression of Nanog in GFP (+) cells were higher than GFP (-) cells significantly both at mRNA and protein level (Figure 2B and 2C, Supplementary S3D and S3E). So did the clone and sphere formation assays, which the GFP (+) cells generated more clones and spheres than GFP (-) cells (Supplementary Figure S3F and S3G).

SUPPLEMENTARY METHODS

Nanog labeled by CRISPR/Cas9 system in HCC cells

The CRISPR/Cas9 plasmid PX330 (Addgene, #42230) was digested with BbSI (New England Biolabs), Nanog-gRNA (Supplementary Table S1) were designed through online tool (http://crispr.mit.edu/) and inserted into the pX330 BbsI cloning site by T4 ligase (Promega). Then it was transformed in Escherichia coli Top10, and plasmids extracted for sequencing.

The homologous arms that up or down of *Nanog* gene termination codon were amplified from human whole genome DNA by KOD FX (TOYOBO), and jointed with 2A-GFP by Gibson clone [2]. Briefly, the fragments of homologous arms were amplified with Nanog-up and Nanog-down primers (Supplementary Table S2) and cloned into pMD19-T simple vector (Takara). Then, it was amplified by Nanog-insert-primers (Supplementary Table S2) to get the *Nanog* homology arm fragment, 2A oligodeoxynucleotides (2A-up/2A-down) were synthesized and annealed before using, and GFP sequence were amplified with GFP primer (Supplementary Table S2); pMD19-T simple vector were amplified with Nanog-vector primers (Supplementary Table S2) to get the clone vector fragment, then all of these fragments were added in Gibson clone buffer (New England Biolabs) to connect into loops and transform to competent Escherichia coli Top10.

Endonuclease enzyme digested and T7E1 assay

The whole genome DNA of 1×10^6 target cells were extracted and amplified with the verify primer 1 (Supplementary Table S1), then the PCR products were purified and digested by T7E1 (New England Biolabs) in 37°C for 1h before detected by 1% agarose gel. For HindIII digested, the DNA were amplified with verify primer 2 (Supplementary Table S1), and the purified PCR product digested by HindIII (New England Biolabs) in 37°C for 1h.

REFERENCES

1. Hadden JM, Declais AC, Carr SB, Lilley DM and Phillips SE. The structural basis of Holliday junction resolution by T7 endonuclease I. Nature. 2007; 449:621-624.
2. Gibson DG, Young L, Chuang RY, Venter JC, Hutchison CA, 3rd and Smith HO. Enzymatic assembly of DNA molecules up to several hundred kilobases. Nature methods. 2009; 6:343-345.

SUPPLEMENTARY FIGURES AND TABLES

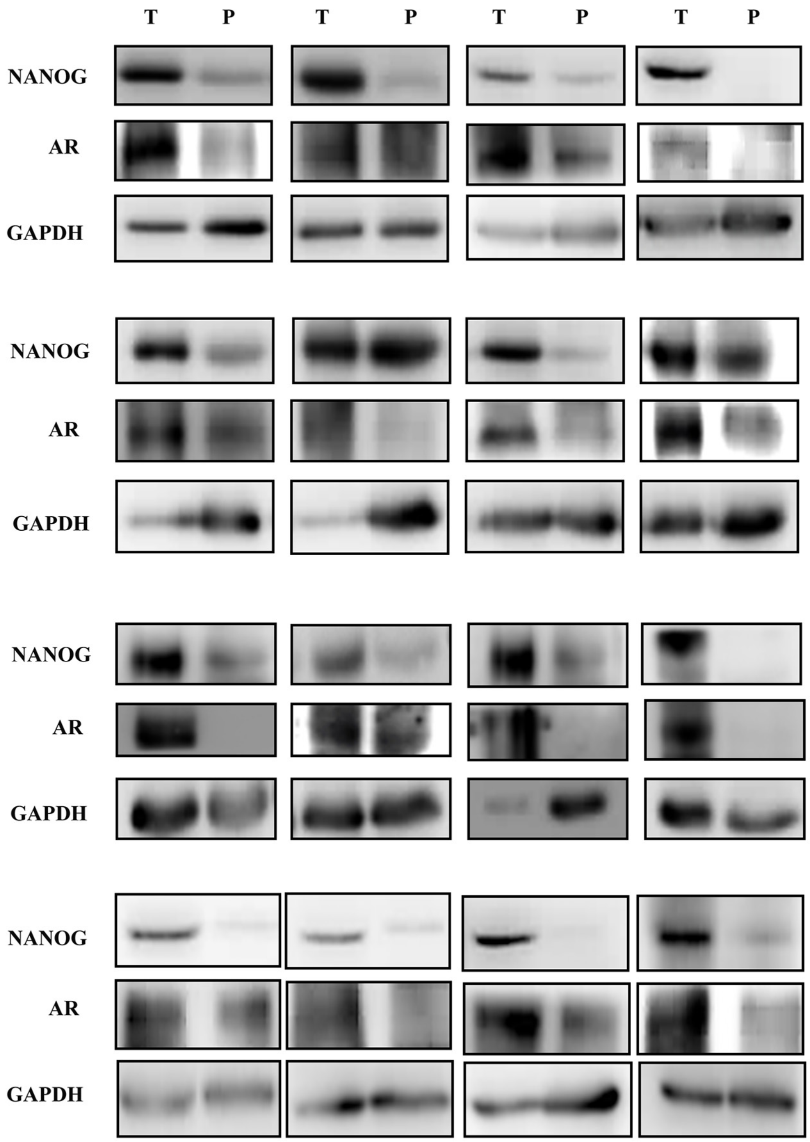

Supplementary Figure S1: AR and Nanog expressed in HCC tissues. The AR and Nanog expression in 16 pairs of hepatocarcinoma and corresponding peritumoral tissues. T: tumor, P: peritumor. GAPDH detected as internal reference.

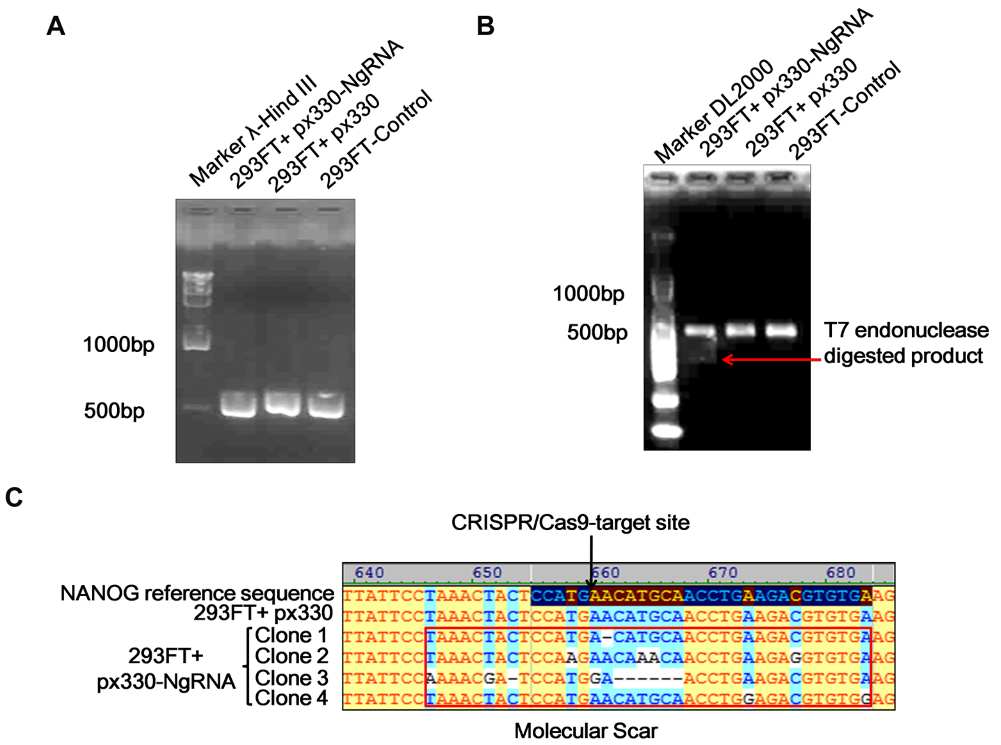

Supplementary Figure S2: CRISPR/Cas9 system targeting verified in 293FT cells. A. The verified PCR products of HEK293FT targeted cells, 293FT-control: the control group (no treated), 293FT+PX330: the group added PX330 vector, 293FT+PX330-NgRNA: the group added PX-330 contained the Nanog-gRNA. **B.** T7 endonuclease digested result of PCR product, the red arrow indicates the digested products. **C.** Sanger sequencing of T7 endonuclease products, the black arrow indicated the Cas9 cut site in *Nanog* gene, the sequence in red frame indicated the "molecular scars" of the repaired sequence after CRISPR/Cas9 cutting.

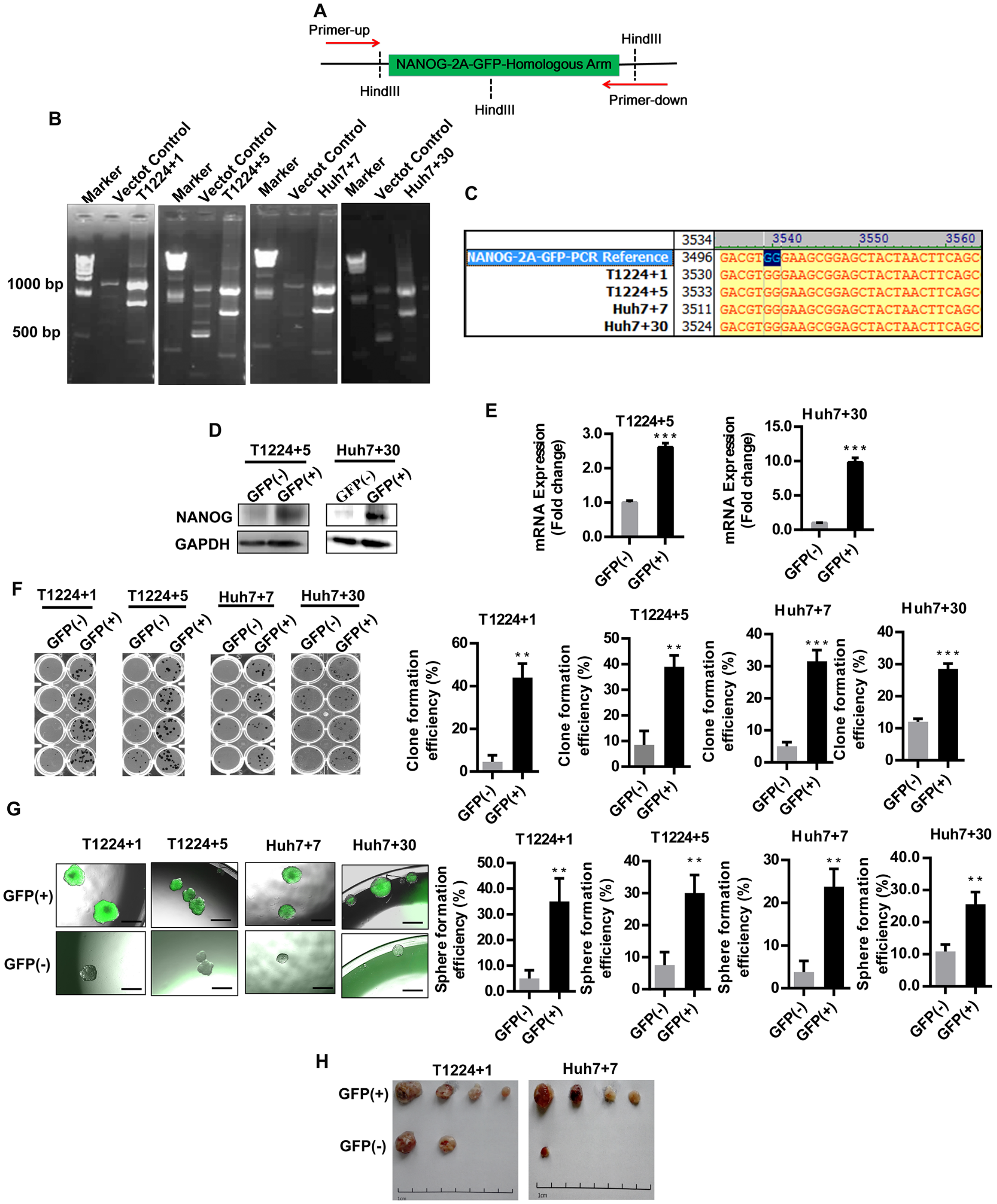

Supplementary Figure S3: Nanog labeled single clone cells verified in vitro and in vivo. A. The schematic of verified PCR and digested products, green frame: the NANOG-2A-GFP homologous fragment contain 2Kb upstream to GFP downstream homologous arms sequence; primer up: the forward primer of verify primer 2 that do not bind to recombination homologous arm vector, down primer: the reverse primer of verify primer 2 that bind to the downstream homologous arm. Black dashed line: λ-Hind III endonuclease digested sites. **B.** The HindIII digested result of four single clone cells PCR products that amplified with the whole genome DNA or vector control (pMD19-T simple vector) by verify primer2. Marker: λ-HindIII-digested. **C.** Sanger sequencing of four single clone PCR products. Dark blue bases: the junction base pairs of Nanog exon4 and 2A-GFP. **D-E.** Protein and mRNA level of Nanog and AR expression in T1224+5 and Huh7+30 single clone GFP (+)/(-) cells. Values are normalized to GAPDH and represented the mean ± SD of triplicate samples. **F-G.** Clone and sphere formation efficiencies of the four single clone GFP (+) or (-) cells, Data was presented as means ± SD of three independent experiments. Scale bar, 200μm. **H.** The subcutaneously transplanted tumors derived from T1224+1 or Huh7+7 GFP (+)/(-) cells in NOD/SCID mice. Each cell type was repeated twice in one mouse and 3 mice per group. $p<0.05$(*), $p<0.01$(**) and $p<0.001$(***).

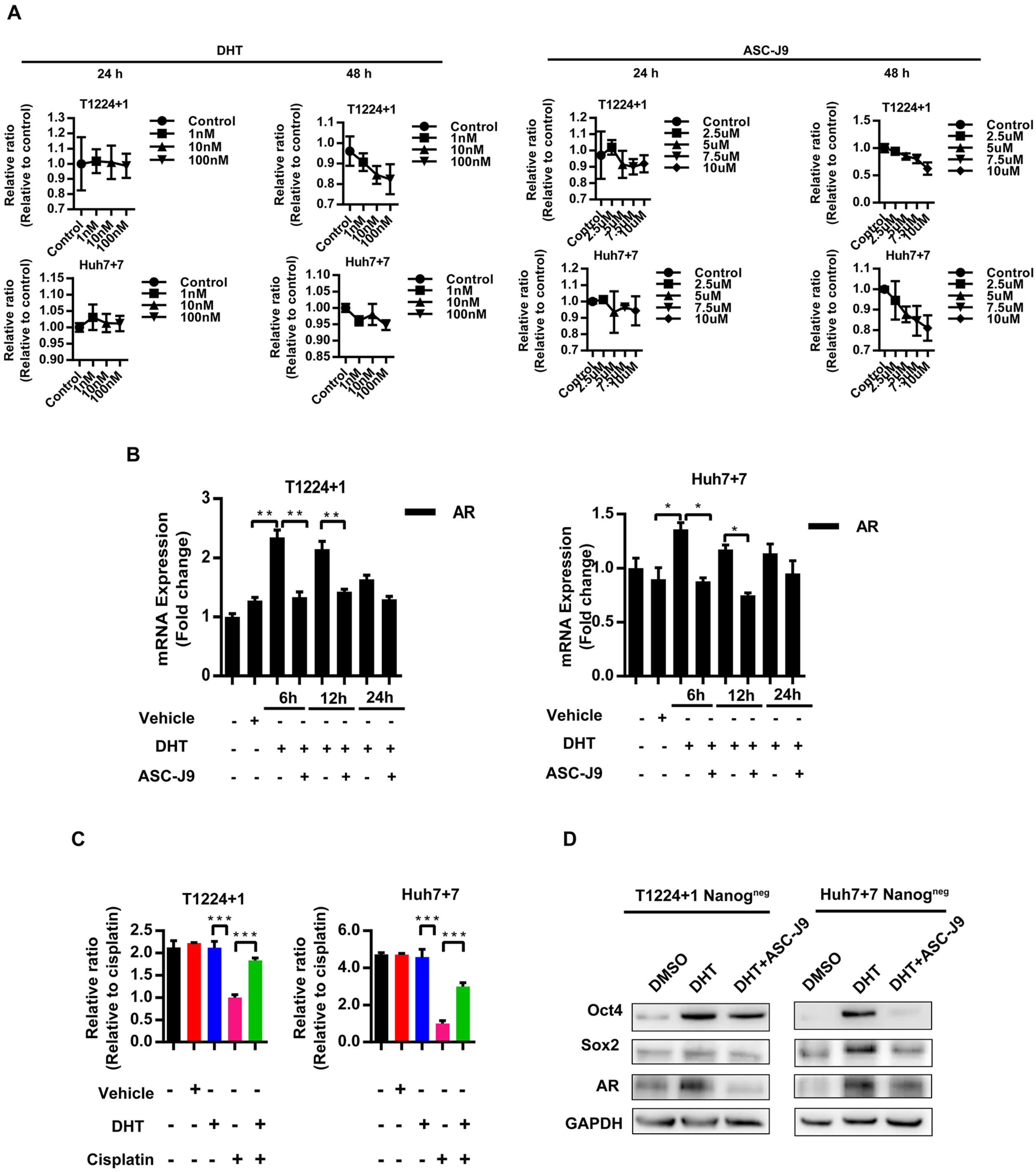

Supplementary Figure S4: Cell viabilities, AR mRNA expressions and stemness verified in cells treated by different compounds. A. Cell viability detected by MTS assay in T1224+1 and Huh7+7 cells treated by DHT or ASC-J9 for 24h and 48h. DHT concentrations were 1nM, 10nM and 100nM, and ASC-J9 as 2.5μM, 5μM, 7.5μM and 10μM, respectively. Data was presented as means ± SD of three independent experiments. **B.** Level of AR mRNA was detected at the different time points after treatment with DHT together with or without ASC-J9 in T1224+1 and Huh7+7 cells. Values are normalized to GAPDH and represented the mean ± SD of triplicate samples. **C.** Cells viability detected by MTS assay in T1224+1 and Huh7+7 cells treated by Cisplatin with or without DHT, cisplatin: 5ug/ml, DHT: 10nM. **D.** Oct4 and Sox2 expression in T1224+1 and Huh7+7 Nanog^{neg} cells treated by DHT with or without ASC-J9. DMSO was vehicle. $p<0.05$(*), $p<0.01$(**) and $p<0.001$(***).

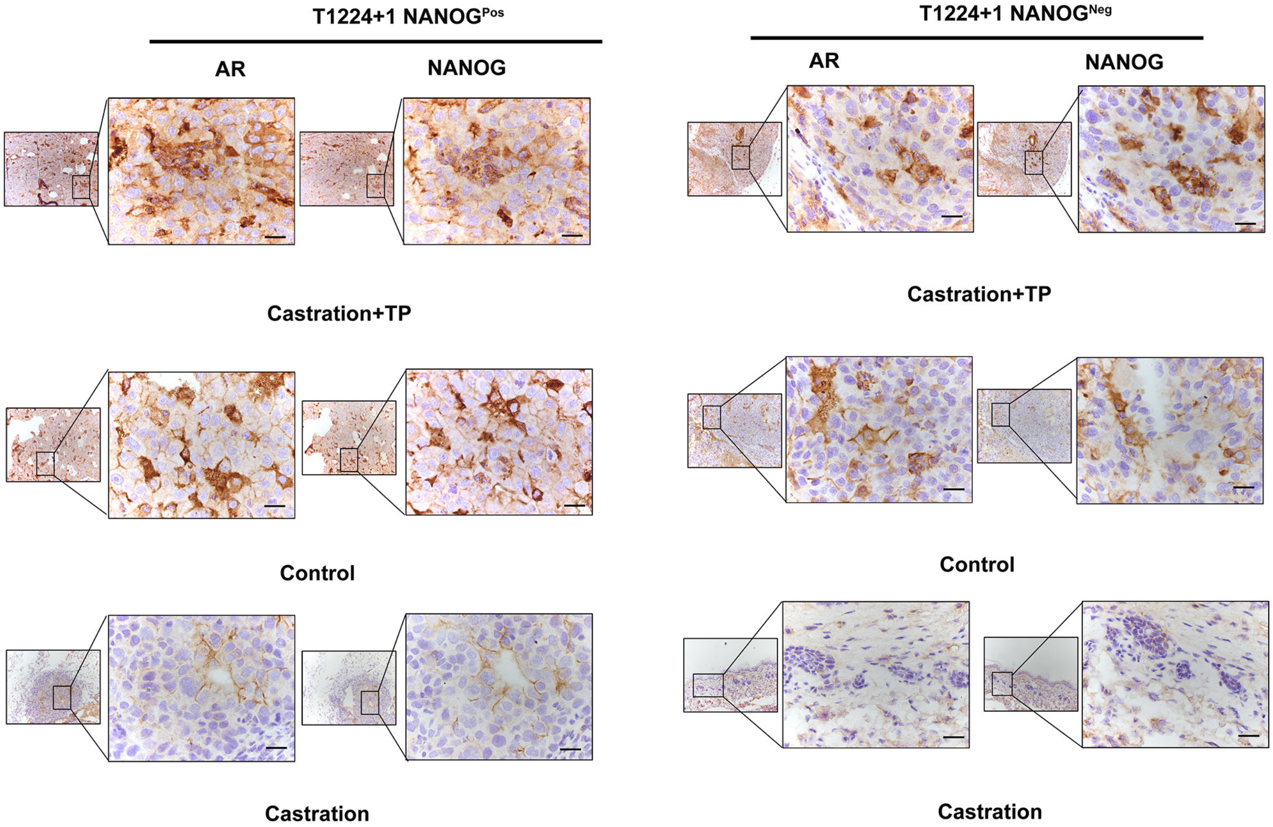

Supplementary Figure S5: Immunohistochemistry stain of T1224+1 cells subcutaneous transplantation tumor sections. Immunohistochemistry staining of AR and Nanog expression in continuous sections from subcutaneous transplanted tumors derived from T1224+1 cell. Scale bars, 100μm.

Supplementary Table S1: The Nanog guide RNA and verify primers

Name	Sequence
**NANOG guide RNA**	CCATGAACATGCAACCTGAAGAC
**Verify Primer 1**	Forward: ACACACAACTCCAGTCACAGACAGTTC<br>Reverse: TTTCTCCAGGAAGATCCAATAGGAAAA
**Verify Primer 2**	Forward: AGAGCCTGTCCCTTTGTTATGTGACTG<br>Reverse: TGTCCAGACTGAAATTGAGTAATATCAGTTTCA CTCATCT

Supplementary Table S2: Primers and oligos for gibson clone

Name	Sequence
**NANOG-up**	Forward: TGAGGTGCTGGTTTTATAGAATCCCCA<br>Reverse: CACGTCTTCAGGTTGCATGTTCATGG
**NANOG-down**	Forward: AGATGAGTGAAACTGATATTACTCAATTTCAG TC<br>Reverse: AACATAATACAGGGCTAGGCTGGTGAC
**NANOG-insert**	Forward: AACTCGGTACGCGCGGATCTTCCAGAGATTTG AGGTGCTGGTTTTATAGAATCCCCAGAA<br>Reverse: GCCAAGTTTGCACGCCTGCCGTTCGACGATAA CATAATACAGGGCTAGGCTGGTGACTCA
**NANOG-vector**	Forward: TGAGTCACCAGCCTAGCCCTGTATTATGTTAT CGTCGAACGGCAGGCGTGCAAACTTGGC<br>Reverse: TTCTGGGGATTCTATAAAACCAGCACCTCAAA TCTCTGGAAGATCCGCGCGTACCGAGTT
**2A-Primer**	Forward: GGAAGCGGAGCTACTAACTTCAGCCTGCTGAA GCAGGCTG<br>Reverse: GCTGAAGCAGGCTGGAGACGTGGAGGAGAACC CTGGACCT
**GFP- primer**	Forward: GAGAACCCTGGACCTGTGAGCAAGGGCGAG<br>Reverse: TGGACGAGCTGTACAAGTAAAGATGAGTGAA ACTGATATT

Supplementary Table S3: Primers for RT-qPCR

Name	Sequence
**NANOG**	Forward: CTCTCCTCTTCCTTCCTCCAT<br>Reverse: TTGCGACACTCTTCTCTGC
**AR**	Forward: TAGCCCCCTACGGCTACA<br>Reverse: TTCCGAAGACGACAAGATGGAC
**GAPDH**	Forward: CACCCACTCCTCCACCTTTG<br>Reverse: CCACCACCCTGTTGCTGTAG

Supplementary Table S4: Primers for ChIP-qPCR

Name	Sequence
**Region 1**	Forward: TGGAAACGTGGTGAACCTAG<br>Reverse: AGTCTCACCAAGGCCATTG
**Region 2**	Forward: CGGCCTCCCAATTTACTG<br>Reverse: TCTAGGTTCACCACGTTTC
**Region 3**	Forward: GGGATAGACAAGAAACCAAAC<br>Reverse: CAACTAGCTCCATTTTCCTC
**Region 4**	Forward: TGGGTTTGGGAATAGGAAGGA<br>Reverse: AGACTACTCCGTGCCCATCT
